# Effect of ultrasonic degradation on the physicochemical property, structure characterization, and bioactivity of *Houttuynia cordata* polysaccharide

**DOI:** 10.1016/j.ultsonch.2025.107331

**Published:** 2025-03-26

**Authors:** Mohammed Mansour, Ramy M. Khoder, Lin Xiang, Lan Lan Zhang, Ahmed Taha, Alsadig Yahya, Ting Wu, Hassan Barakat, Ibrahim Khalifa, Xu Xiaoyun

**Affiliations:** a College of Food Science and Technology, Huazhong Agricultural University, Wuhan 430070, China; b Key Laboratory of Environment Correlative Food Science (Huazhong Agricultural University), Ministry of Education, China; c Hubei Key Laboratory of Fruit & Vegetable Processing & Quality Control, Huazhong Agricultural University, Wuhan, 430070, China; d Desert Research Center (DRC), Matariya, Cairo, Egypt; e Department of Food Science, Faculty of Agricultural, (Saba Basha), Alexandria University, Alexandria 21531, Egypt; f Department of Food Science and Human Nutrition, College of Agriculture and Food, Qassim University, Buraydah 51452, Saudi Arabia; g Food Technology Department, Faculty of Agriculture, Benha University, Moshtohor, Toukh 13736, Egypt

**Keywords:** *Houttuynia cordata* polysaccharide, Ultrasonication, Structure characterization, Antioxidant activity, Hypoglycemic activity, Polyphenols

## Abstract

This study aimed to evaluate the influence of ultrasonic degradation on *Houttuynia cordata* polysaccharide (HCP) physicochemical properties, structure characterization, and bioactivities. The results indicated that the ultrasonic degradation could significantly decrease HCP’s molecular weight (MW). Total polysaccharide, uronic acid content, solubility, and thermal stability of HCP increased gradually with the increase in ultrasonication power. Fourier transform infrared (FTIR) and Nuclear magnetic resonance spectroscopy (NMR) spectra proved that the primary structure of HCP had not been changed via ultrasonic degradation. Antioxidant and hypoglycemic activity results confirmed that ultrasonication enhanced the ability to scavenge free radicals (DPPH, ABTS, and OH) and improved α-glycosidase and α-amylase inhibition with the increase of ultrasonic power, which was increased in order HCP <U200 < U400 < U600. The degraded HCP produced via 600 W presented the best physicochemical properties and bioactivities. U600, α-amylase and α-glycosidase inhibition activities were 43.80 ± 0.68 and 83.28 ± 2.56 %, which were higher than those of native HCP 38.40 ± 0.53 and 65.67 ± 0.54 %, respectively, at a concentration of 10 mg/mL HCP solution. These results suggested that ultrasonication could be used as a green method for polysaccharide degradation and showed potential application for enhancing polysaccharide bioactivities for functional foods and pharmaceutical applications.

## Graphical abstract

**Figure:**
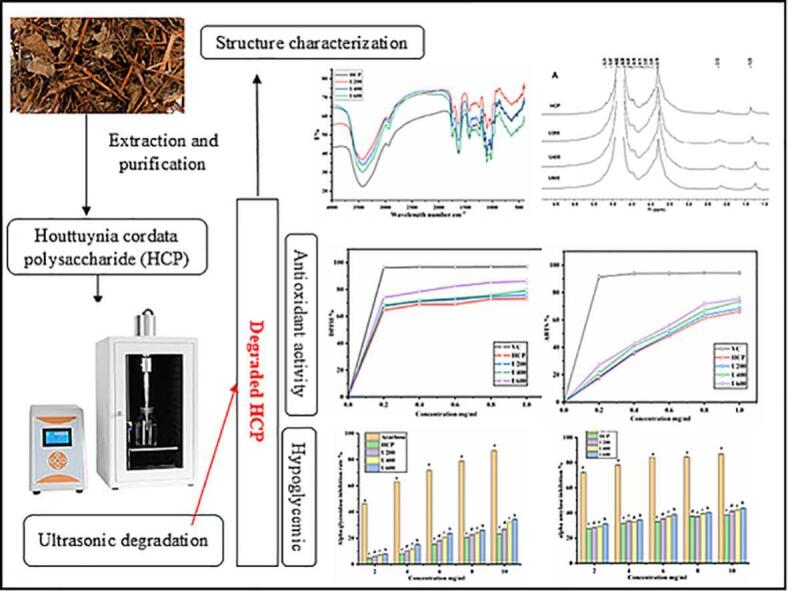

## 1 Introduction

*Houttuynia cordata,* a medicinal plant of the *Saururaceae* family, grows in humid soil and warmer regions. It is extensively prevalent throughout Asia, especially in China, Japan, Korea, and Southeast Asia [1]. In China, *Houttuynia cordata* is recognized for its medicinal properties, serving a crucial function in conventional healthcare and disease management [2]. It is commonly used to treat lung abscesses, colds, fever relief, swelling reduction, and urinary promotion [3].

One of the most effective bioactive components of *Houttuynia cordata* is a polysaccharide, known as an acidic heteropolysaccharide mainly composed of arabinose, galactose, xylose, glucose, galacturonic acid, glucuronic acid, rhamnose, and mannose [4], which exhibited many interesting bioactivities such as antioxidant, anti-inflammatory, and immunity regulation [5]. Notably, *Houttuynia cordata* polysaccharide (HCP) exhibits significant antioxidant activity, which likely contributes to its anti-inflammatory effects by mitigating oxidative stress and suppressing the generation of reactive oxygen species (ROS) [6]. On the other hand, HCP has an immunomodulation activity via increasing the secretions of macrophage inhibitory protein-1 (MIP-1), macrophage inhibitory protein-1 (MIP-1), tumor necrosis factor- (TNF-α), and interleukin-1(1L-1) [7]. Moreover, HCP has a quite effect as antidiabetic, as reported by Liu et al. [4] while studying the effect of *Houttuynia cordata* stem polysaccharide for inhibition α- amylase and α-glycosidase. Therefore, there is an interest in discovering a new method that could enhance the biological activity of HCP.

As is known, polysaccharide's physiochemical properties and functional characterization are closely linked to their structure properties, such as molecular weight, branching degree, and configuration [8]. Many studies have proved that polysaccharide's molecular weight decreased after various degradation methods. Although the monosaccharide compositions and functional group of polysaccharides were similar before and after degradation, they significantly improved the biological activity [9,10]. Polysaccharide degradation is performed *via* different physical, chemical, and biological methods. Among them, we will use the physical modes of ultrasonic degradation.

Ultrasonication is considered an effective and green physical method to improve the bioactivities of polysaccharides and could be used at a wide range of power to produce different degraded polysaccharides [11]. Ultrasonication depends on acoustic cavitation through various mechanisms; the mechanical energy created from cavitation results in irreversible chain disruption. The disruption of a polysaccharide chain could occur randomly or in the midpoint of the chain, depending on the structure and nature of the polysaccharide [12]. The polysaccharide bioactivity was improved after the ultrasonication, as mentioned by Li et al. [13], who proved that the ultrasonication treatments improved the physiochemical properties of polysaccharides produced by *Chaetomium globosum* fungi and increased the antioxidant and antibacterial activity. Xu *et al.* [14] confirmed that the degradation of black currant polysaccharides via different ultrasonication treatments decreased the particle size and molecular weight of polysaccharides, increasing the antioxidant and antidiabetic effect of degraded polysaccharides. Therefore, ultrasonication can be considered a promising method for polysaccharide modification and a beneficial green method to conjugate polysaccharides in pharmaceutical and food processing [15].

Based on the literature review, few studies have been reported on the effects of sonication on the structure of polysaccharides and their functional characteristics. However, to date, there have been no reports on the impact of ultrasonic degradation on HCP physicochemical properties and bioactivity. We hypothesize that ultrasonic degradation will significantly change HCP physicochemical properties, decrease molecular weight, improve solubility and thermal stability, and enhance antioxidant and hypoglycemic activities. Therefore, this study distinguished the influence of ultrasonication treatment on the physicochemical properties, structure characterization, antioxidant activity, and *in vitro* hypoglycemic activity of HCP. This study's results could help provide valuable information about the effects of ultrasonic degradation on polysaccharide bioactivities and enhance the utilization value and application of HCP.

## 2 Material and methods

### 2.1 Materials

*Houttuynia cordata* plant was planted in Lianghe town, Dangyang City, Yichang City, Hubei Province; Monosaccharides (Mannose, glucose, galactose, fucose, arabinose, xylose, glucuronic acid, and galacturonic acid), and Dextran standards were purchased from Aladdin Chemistry Co., Ltd. (Shanghai, China). α-amylase and α-glycosidase enzymes were purchased from (Sigma-Aldrich, St. Louis., MO, US). 4-Nitrophenyl-*α*-D glucopyranoside (*p*-NPG) and acarbose were obtained from Shanghai Yuanye Biotechnology Co. Ltd. All chemical reagents were of analytical grade.

### 2.2 Extraction of *Houttuynia cordata* polysaccharide

*Houttuynia cordata* (HC) leaves and stems were grounded via an electric grinder and sized with 60 mm sieves. HC powder was degreased by soaking in 95 % ethanol for 12 h, then filtrated and dried at room temperature. The extraction of polysaccharides was performed using the hot water extraction method. About 50 g of degreased HC powder was dissolved in 1L distilled water (DW) and heated at 95 °C for 2 h. The extraction procedure was repeated twice; the supernatant was collected via centrifuge at 8000g for 10 min and condensed to 1/5 of the whole solution using a rotary evaporator. The condensed polysaccharide solution was precipitated using ethanol at a ratio of 1:4 at 4 °C for 12 h. After that, the precipitate polysaccharide was collected by centrifuge and filtration, washed at least twice with ethanol, and dried at low temperature to produce crude HCP.

### 2.3 Isolation and purification of HCP

HCP was isolated and purified using the three-phase partitioning method according to Yan et al. [16]; with some modification, the crude polysaccharide was dissolved in DW to reach a concentration of 10 mg/mL and adjusted pH to 6. At 50 mL centrifuge tubes, the ammonium sulfate was added at a concentration of 25 % w/v of polysaccharide solution and vortex until it was totally dissolved. After that, t-butanol was added at the same volume of polysaccharide solution, and the mixture was stirred at 35 °C for 30 min, centrifuge the mixture at 2800g for 10 min to separate the three phases. The upper layer contained t-butanol with organic soluble material, the middle layer contained free protein, and the lower layer contained polysaccharide solution with a high content of ammonium sulfate, which was separated carefully and dialyzed via (FDM403 3.5 kDa) against DW for 72 h to remove the salts. After dialysis, the polysaccharide solution was concentrated and freeze-dried to produce purified HCP.

### 2.4 Ultrasonication degradation of HCP

Ultrasonic degradation was operated via an ultrasound cell grinder (Sonics, Uibro cell, USA), and the polysaccharide solution with a concentration of 6 mg/mL was subjected to the reaction vessel (a 100 mL glass beaker) for ultrasonic treatment at different power levels of 200,400 and 600 W with 1sec on and 1sec off for 30 min using a 6 mm probe always immersed at the middle of the solution. The temperature was maintained using an ice bath. After the ultrasonication treatment, the produced solution was dialyzed for 48 h and freeze-dried to produce different degraded HCPs, named U-200, U-400, and U-600.

### 2.5 Physicochemical properties

#### 2.5.1 Chemical composition analysis and solubility

Total carbohydrates were measured via the phenol–sulfuric acid method, according to Dubois et al. [17]. About 1 mL of HCP was subjected to a 10 mL tube with a cap, and 50 µL of 80 % phenol solution was added and vortexed. Then, 5 mL of concentrated sulfuric acid was added, and vortexed the mixture. The produced mixture was boiled for 20 min, cooled to ambient temperature, and the absorbance was measured at 490 nm via UV–visible spectrophotometer (Thermo Scientific, UK); D-glucose was used as a standard. Protein content was measured via the Bradford method using bovine serum albumin as a standard. Briefly, 1 mL of polysaccharide solution was subjected to a 10 mL capped tube, and 5 mL of protein reagent was added; the mixture was vortexed, and the absorbance was measured at a wavelength of 595 nm after 30 min via UV–visible spectrophotometer. Uronic acid content was measured using *meta*-hydroxydiphenyl reagent according to Blumenkrantz et al. [18] with some modification and using glucuronic acid as a standard. Briefly, 1.2 mL of borate–sulfuric acid solution was kept in an ice bath, and 0.2 mL of HCP solution (600,800 and 1000 µg/ mL) was added. The resulting mixture was vortexed, cooled, and heated in bath water at 100° C for 5 min. After cooling the reaction mixture via an ice bath, 20 µL of meta- hydroxydiphenyl (0.15 % w/v) was added. The absorbance of the produced solution was measured at 520 nm. The solubility of HCP with and without ultrasonication was determined according to the Wu et al. [19] method with some modification; about 50 mg of HCP (W0) was dissolved in distilled water and vortexed for 5 min. Then centrifuged at 6000 xg for 10 min, the supernatant was collected and dried via oven at 105 °C until weight constant (W1). Solubility was calculated via Eq. (1).

(1) S%=W1/W0×100

#### 2.5.2 Molecular weight

The molecular weights of native and sonicated HCP were assessed according to Zongo et al. [20] with some modifications using a size exclusion chromatography system integrated with multi-angle laser light scattering (SEC-MALLS) (Dawn Helios II, Wyatt Technology Co., Santa Barbara, CA, USA) and a refractive index detector (OPTILAB Trex, Wyatt Technology Co., Santa Barbara, CA, USA). 6 mg/mL of HCP solution in 0.1 M NaNO_3_ was prepared and filtered via a 0.45 µm syringe filter. Subsequently, 0.5 mL was injected into a chromatographic system connected in series to an OHpak SB-G guard column (6 × 50 mm^2^) and OHpak SB-806 HQ columns (8 × 300 mm^2^) from Shodex Co., Tokyo, Japan. The flow rate of the 0.1 M sodium nitrite mobile phase was established at 0.4 mL/min, and the column temperature was maintained at 25 °C. Dextran 40 kDa served as a standard for normalizing the obtained data. The results were evaluated using Astra software version 6.1.2.

#### 2.5.3 Monosaccharide composition

Monosaccharide composition was determined via gas chromatography Agilent 6890 system GC (Agilent Technologies, Palo Alto, CA, USA) according to Xie et al. [21] with minor modification; about 2 mg of HCP was hydrolyzed in a sealed ampoule with 1 mL of trifluoroacetic acid solution 2 M at 110 °C for 4 h. The hydrolyzed polysaccharide was evaporated *via* an N_2_ blowing device, and then 2 mL methanol was added to co-evaporate the residual acid, which was repeated twice. After removing the acid and methanol, 1 mL pyridine, 10 mg hydroxylamine hydrochloride, and 2 mg fucose were added to the residue and heated at 95 °C for 30 min in a water bath. Acetic anhydride was added, and the mixture was acetylated at 95 °C for 30 min. The solvent was evaporated, yielding acetylated monosaccharides, which were subsequently dissolved in chloroform for analysis.

### 2.6 Structural characterization

#### 2.6.1 Fourier-transform infrared spectroscopy (FTIR)

FTIR analysis of HCP with and without ultrasonication was performed via FTIR Nicolet 470 (Thermo Fisher Scientific, USA). About 5 mg of polysaccharide was grounded with 100 mg KBr and compressed to produce a KBr disk. The spectra were recorded at 4000–400 cm^−1^, and the spectra were processed via Omnic software.

#### 2.6.2 Nuclear magnetic resonance spectroscopy (NMR)

The freeze-dried HCP and sonicated HCP were exchanged with D2O two times, followed by lyophilization, and about 50 mg was dissolved in 1 mL D2O and loaded into the NMR tube. ^1^H NMR and ^13^C NMR spectra were recorded via (Burker Avance 600 MHz Neo SPEC).

#### 2.6.3 Thermogravimetric analysis (TGA)

TGA of HCP with and without ultrasonication was performed according to Li et al. [22] with some modification using a thermo-gravimetric analyzer (STA 449 Jupiter F5, Netzsch, Germany). About 20.0 mg sample was heated within a 30–600 °C temperature range at a heating rate of 10 °C/min under a nitrogen flow of 50 mL/min.

#### 2.6.4 X-ray diffraction (XRD)

HCP crystal structure with and without ultrasonic degradation was analyzed via X-ray diffraction (XRD, D8 Advance X-ray diffractometer, Burker, Germany) with Cu-Kα radiation (λ = 0.154 nm) at 40 Kv and 40 mv. The scattering measurements were obtained at an angle range from 5° to 55° at a scanning velocity of 5°θ / min.

#### 2.6.5 Scanning electron microscope (SEM)

The microstructure of freeze-dried HCP with and without ultrasonication treatment was captured via SEM. The samples were mounted on a metal stab and sputtered with gold film, and the samples were examined by an SEM device (JEOL JSM − 6390 LV, Oxford instruments) at a 5 kV acceleration voltage.

#### 2.6.6 Atomic force microscopy (AFM)

The molecular morphology of HCP with and without ultrasonication was observed *via* AFM (Dimension icon, Burker, NanoScope, Germany). Samples solutions were diluted to 2.5 µg/mL before 5 µL dropped on a mica surface and dried at room temperature. After that, the mica surface was fixed to the AFM, and the images were captured via tapping mode.

#### 2.6.7 Congo red analysis

The Congo red technique was used to determine the triple helix of HCP with and without ultrasonication, according to Liu et al. [23]. Briefly, 2 mL of Congo red solution (80 µM) was combined with HCP solution (2 mg/mL), followed by the addition of different concentrations of NaOH (0–0.5 M). Congo red solution was used as control. The maximal absorption wavelength was determined by UV–visible spectrophotometer (Thermo Scientific, UK) between 400 and 700 nm.

### 2.7 *In vitro* antioxidant activity

#### 2.7.1 2,2-diphenyl-1-picrylhydrazyl radical (DPPH) scavenging activity

HCP with and without ultrasonication DPPH radical activity was done according to the Liu et al. [24] method with some modification. DPPH at a concentration of 0.1 mM was prepared in an ethanol solution. A polysaccharide sample was generated at varying concentrations of 0.2, 0.4, 0.6, 0.8, and 1 mg/mL. In a 96-well plate, 100 µL of various concentrations was added, followed by 100 µL of DPPH solution. The mixture was then incubated in the dark for 30 min, after which the absorbance was measured at a wavelength of 517 nm using (Multiskcan SkyHigh, Thermo Fisher Scientific Co., Ltd., Shanghai, China). The DPPH radical scavenging activity was quantified using Eq. (2).

(2) DPPHradicalscavengingactivity%=(A0-(As-A1))/A0×100

A0 represents the absorbance of the DPPH solution with distilled water (DW) in place of the sample, As indicates the absorbance of the tested sample with the DPPH solution, and A1 represents the absorbance of the sample with ethanol as the background.

#### 2.7.2 2,2-azinobis-(3-ethylbenzothiazoline-6-sulfonic acid (ABTS) radical scavenging activity

According to Cheng et al. [25], ABTS radical scavenging activity was measured with some modification. ABTS solution was mixed with 7 mM ABTS radical scavenging solution with 140 mM potassium persulfate solution and kept the mixture in the dark overnight. ABTS solution was diluted via phosphate buffer pH 7.4 (1:80) until the absorbance of ABTS solution was around 0.7 nm. Different Vitamin C and HCP concentrations with and without ultrasonication were prepared 0.2,0.4,0.6,0.8, 1 mg/mL and loaded into 96 well plate with volume 20 µL, ABTS solution 180 µL was added. The mixture was kept for 5 min. The absorbance of the mixture was obtained at wavelength 734 nm by (Multiskcan SkyHigh, Thermo Fisher Scientific Co., Ltd., Shanghai, China), and ABTS scavenging activity was calculated according to the formula (3).

(3) ABTSradicalscavengingactivity%=(A0-AS)/A0×100

Where A0 is the absorbance of ABTS solution when DW replaces the sample, and AS is the absorbance of the tested sample and ABTS solution.

#### 2.7.3 Hydroxyl radicle (OH) scavenging activity

Hydroxyl radicle scavenging activity was performed according to Wang et al. [26] with slight modification. In brief, HCP with and without ultrasonication treatment with different concentrations (0.2,0.4,0.6,0.8 and 1 mg/mL) was mixed with FeSO_4_ 0.7 mmole, H_2_O_2_ 10 mmole, and salicylic acid ethanolic solution 0.7 mmole. The final mixture was incubated at 37 °C for 30 min, and the absorbance was measured via (Multiskcan SkyHigh, Thermo Fisher Scientific Co., Ltd., Shanghai, China) at 510 nm. Vitamin C was used as a positive control, and the hydroxyl radicle scavenging was calculated via Eq. (3).

(4) OHscavengingactivity%=(A0-(As-A1))/A0×100

Where A0 is the absorbance when the tested sample was replaced via DW, As is the absorbance of the tested sample, and A1 is the absorbance of the tested sample when DW replaced H_2_O_2_.

### 2.8 *In vitro* hypoglycemic activity

#### 2.8.1 α-amylase inhibition assay

The inhibition of α-amylase activity was determined according to Deng et al. [27] with some modification. Approximately 1 mL of α-amylase enzyme solution at a concentration of 0.5 mg/ml, prepared with 0.1 mM phosphate buffer at pH 6.9, was combined with 1 mL of polysaccharide solution at varying concentrations (2, 4, 6, 8, and 10 mg/ml) and acarbose as a positive control. Subsequently, 1 mL of starch solution (2 mg/mL) was introduced, and the mixture was incubated at 37 °C for 20 min. The reaction was terminated by adding 2 mL of dinitro salicylic acid (DNS) reagent (1 % DNS, 12 % potassium sodium tartrate), then boiling the mixture for 5 min in a water bath. The absorbance was measured by (Multiskcan SkyHigh, Thermo Fisher Scientific Co., Ltd., Shanghai, China) at 540 nm after cooling to room temperature. Acarbose was a positive control, and the inhibition of α-amylase activity was calculated using the specified Eq. (4).

(5) α-amylaseinhibitionrate%=1-((A1-A2)/(A3-A0))×100

A1 represents the absorbance of the examined material combined with PBs, α-amylase, starch, and dinitrosalicylic acid (DNS). A2 represents the absorbance of the examined material combined with phosphate-buffered saline (PBS), starch, and DNS. A3 represents the absorbance of PBs combined with α-amylase, starch, and DNS. A0 represents the absorbance of PBs + starch combined with DNS.

#### 2.8.2 α-glycosidase inhibition assay

The inhibition of α-glycosidase was performed based on the method of Xu et al. [28] with some modifications. In a 96-well plate, 25 µL of various concentrations of polysaccharide solution (2, 4, 6, 8, and 10 mg/mL) was dispensed, followed by the addition of 25 µL of α-glycosidase enzyme (0.25 U/mL). The mixture was incubated at 37 °C for 15 min, after which 50 µL of PNPG was added and incubated at 37 °C for an additional 15 min. Subsequently, 100 µL of Na_2_CO_3_ (0.1 M) was added, and the absorbance was measured at 405 nm via (Multiskcan SkyHigh, Thermo Fisher Scientific Co., Ltd., Shanghai, China). Acarbose served as the positive control, and the α-glycosidase inhibitory activity was quantified using Eq. (5).

(6) α-glycosidaseinhibitionactivity%=((A0-Ab)-(As-A1))/((A0-Ab))×100

where A0, is the absorbance when the tested sample is replaced with DW. Ab, is the absorbance of an equivalent amount of PBs and α-glycosidase. As, is the absorbance of the tested sample. A1, is the absorbance of the tested sample without α-glycosidase enzyme.

### 2.9 Statical analysis

All experiments were performed at least triplicates. Variances analysis was operated via one-way ANOVA using IBM SPSS (version 22, IBM, Chicago, IL, USA), and significant differences were determined at P < 0.05. Data presented as mean ± standard deviation.

## 3 Result and discussion

### 3.1 Extraction and purification of HCP

**Fig. 1:**
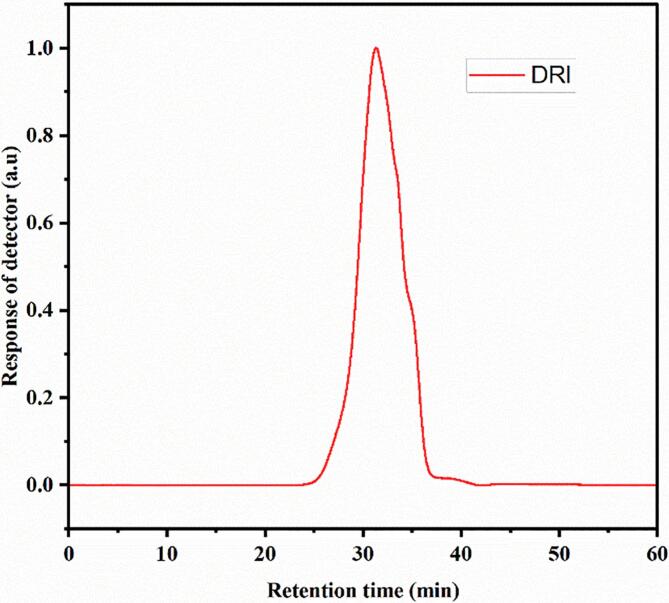
Size exclusion chromatography-multi-angle laser light scattering (SEC-MALLS) DRI chromatogram of HCP.

The crude HCP was obtained via the hot water extraction method followed by purification using the three-phase partitioning method (TTP), which is considered a green method that can produce high-yield and purity polysaccharides. At the same time, it has many advantages, such as being inexpensive, simple, rapid, and efficient [16]. Our results confirmed that the purified HCP purity was increased from 20 ± 1.15 to 88.31 ± 0.42 % w/w, and the protein content of HCP was decreased from 2.274 ± 0.13 to 0.502 ± 0.05 % before and after TPP, respectively. The produced polysaccharide and ultrasonicated treatment presented one peak with good symmetry through gel permission chromatography, indicating homogeneity, as shown in Fig. 1.

### 3.2 Effect of ultrasonication on HCP physicochemical properties

#### 3.2.1 Total polysaccharide, uronic acid content, and solubility

As shown in Table 1, the total polysaccharide content of ultrasonicated HCP was increased with the increase of ultrasonication power. The highest polysaccharide content was for U600 treatment, about 96.54 ± 0.01 %, implying that ultrasonication can increase the polysaccharide content and decrease the impurity content. This could be related to the breakdown of glycosidic bonds of polysaccharides during sonication treatment [14]. Uronic acid content was increased from 23.31 + 0.25 to 26.67 ± 0.01 % for HCP and U600, respectively. In addition, the solubility of ultrasonic degraded HCP was increased with the increase of ultrasonic power, and this phenomenon was related to the decrease in molecular weight. These results were probably due to the scission of the polysaccharide chain, exposure to ultrasonication, and increased free uronic acid from polysaccharides. These results were on the same trend as a result obtained by Wu et al. [19], who studied the effect of ultrasonication on the characterization and bioactivities of *Panax notoginseng* flower polysaccharide and found that ultrasonication treatment can increase the polysaccharide and uronic acid content of polysaccharide solution.

**Table 1:** Physiochemical properties of HCP, U200, U400, and U600.

Property	HCP	U200	U400	U600
Total polysaccharide %	88.31^a^ ± 0.42	90.08^b^ ± 0.11	94.25^c^ ± 0.62	96.54^d^ ± 1.01
Uronic acid content %	23.31^a^ ± 0.25	24.12^b^ ± 0.32	25.61^c^ ± 0.02	26.67^d^ ± 0.01
Solubility %	75.08^a^ ± 2.1	81.31^b^ ± 1.23	83.83^c^ ± 1.12	85.13^d^ ± 1.91
Molecular weight × 10^5^ Da	4.923	3.729	2.574	1.821
Monosaccharide composition (%)				
Mannose	9.06	8.45	9.04	8.74
Rhamnose	10.99	11.21	10.93	11.32
Glucuronic acid	51.66	45.92	44.74	43.42
Galacturonic acid	3.3	3.53	3.04	2.84
Glucose	5.68	7.04	6.92	8.23
Xylose	14.86	18.43	17.8	21.28
Galactose	1.37	1.88	3.05	1.27
Arabinose	3.06	3.5	4.45	2.85

^a–d^ Means with different letters in row different significantly (p < 0.05).

#### 3.2.2 Molecular weight

The average molecular weight of HCP was decreased from 4.923 × 10^5^ for native HCP to 3.729 × 10^5^ (U200), 2.574 × 10^5^ (U400), and 1.821 × 10^5^ Da (U600) after sonication at 200, 400 and 600 W, respectively as presented in Table.1. The polysaccharide properties were altered by the shear forces generated via cavitation bubbles produced by ultrasonic waves. These shear forces typically promote the dispersion of polysaccharide aggregates, resulting in a reduction in molecular weight. These results suggested that the higher ultrasonication power presented a higher degradation of the polysaccharide chain. Our results were on the same trend as Yuan et al. [15], who studied the effect of ultrasonication degradation on *sargassum pallidum* polysaccharide and found that the molecular weight of polysaccharide was decreased with the increase of ultrasonic power.

#### 3.2.3 Monosaccharide composition

**Fig. 2:**
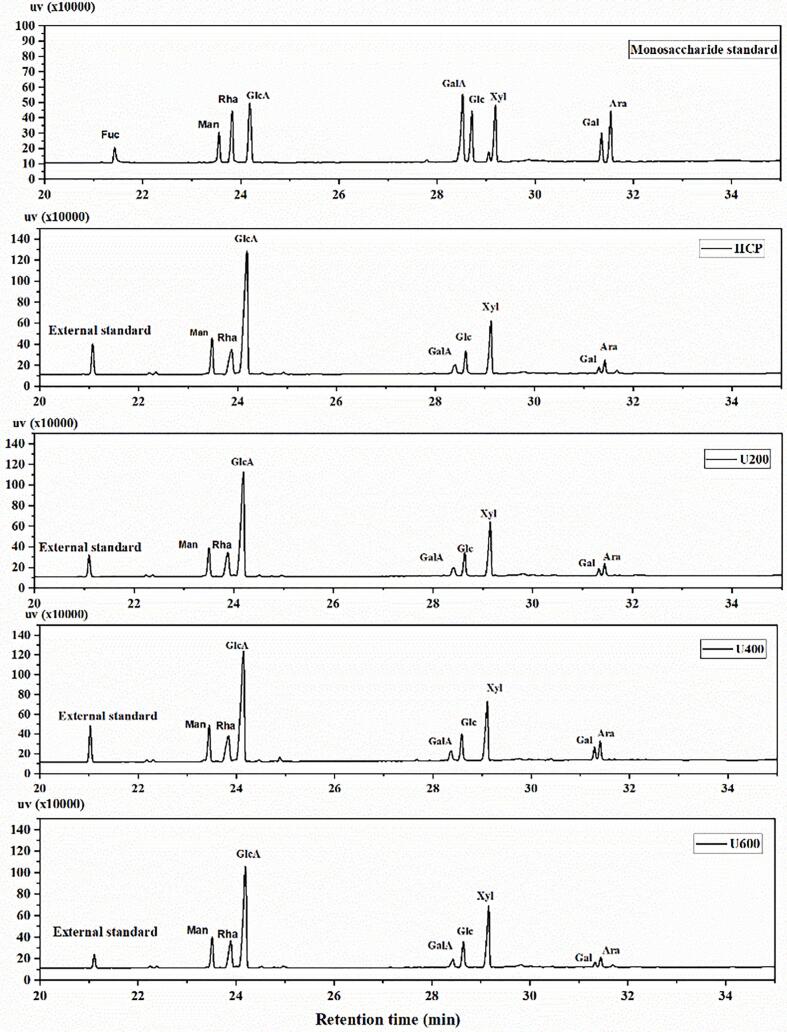
GC chromatogram of monosaccharide standard, HCP, and ultrasonic degraded HCP (U200, U400, U600). (Man: Mannose, Rha: Rhamnose, Glu A: Glucuronic acid, Gal A: Galacturonic acid, Glu: Glucose, Xyl: Xylose, Gal: Galactose, Ara: Arabinose and external standard Fuc: Fucose.

HCP with and without ultrasonic treatment presented the same monosaccharide composition as shown in Fig. 2, which contains 8 monosaccharides, mannose, rhamnose, glucuronic acid, galacturonic acid, glucose, xylose, galactose, and arabinose. The three degraded fractions (U200, U400, U600) contain the same monosaccharide, but there is a slight change in the molar ratio, as shown in Table 1. Our findings confirmed that ultrasonication treatments do not affect the monosaccharide composition of HCP. These results were similar to the result obtained by Cheung et al. [29], who studied the effect of ultrasonication degradation on curdlan polysaccharide using an alkaline solution and found that the ultrasonication treatment had a slight impact on the molar ratio of monosaccharide without any change in the whole monosaccharide composition. This result matched the FTIR and NMR results, confirming the stability of the polysaccharide structure after ultrasonication treatments.

### 3.3 Structural characterization

#### 3.3.1 FTIR

**Fig. 3:**
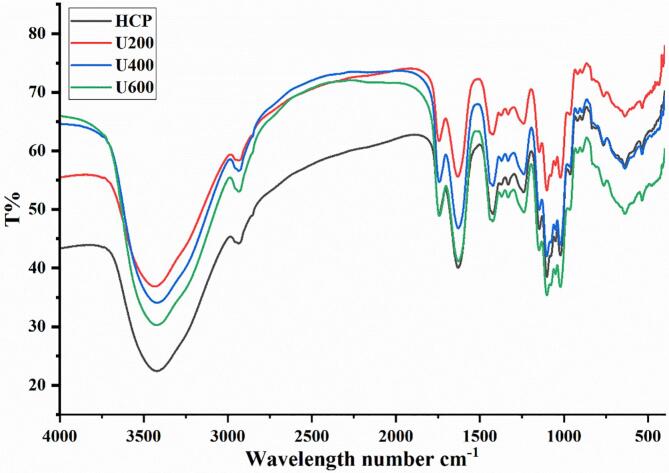
FTIR spectra of HCP and ultrasonic degraded HCP.

FTIR is considered an effective technique for measuring the functional group of the chemical structure of different materials [30]. FTIR spectra of HCP with and without ultrasonication displayed typically characteristic peaks of polysaccharide at the range of 4000–500 cm^−1^ as presented in Fig. 3. The peaks around 3425, 2934, and 1744 cm^−1^ represented O—H, C—H, and C = O vibrations respectively [31]. The peaks around 1630 and 1425 cm^−1^ were attributed to the absorption of the deprotonated carboxylic group, which proved the presence of uronic acid[32], and these findings matched the result of uronic acid content. The peaks appeared at 1102 cm^−1^ and 1018, confirming the presence of pyranose C—O—C and C—O—H [33]. The absorbance peaks band at 890 cm and 829 indicated that HCP was composed of α and β glycosidic bonds [34]. The absorbance peak at 1243 cm is related to the C—O or C—N group, responsible for HCP's acidity [35]. These results showed that the main functional groups had no noticeable changes due to the ultrasonic degradation. It was reported that both the ultrasonic degraded black currant fruit polysaccharide and the native polysaccharide had the same functional groups [14], which matched our results.

#### 3.3.2 TGA analysis

**Fig. 4:**
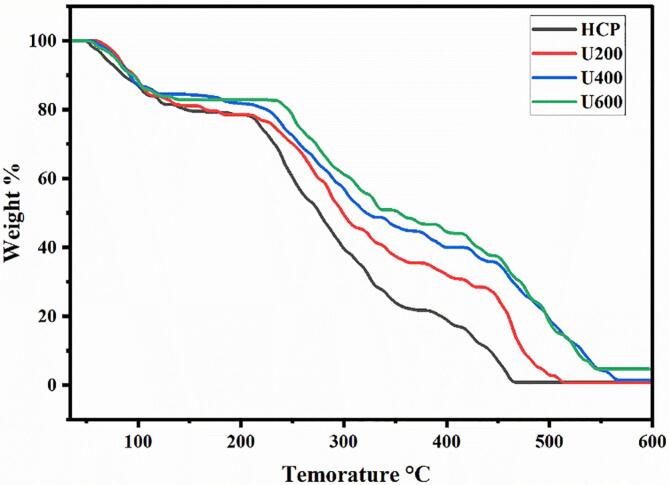
TGA thermogram of HCP and ultrasonic degraded HCP.

To figure the effect of ultrasonic degradation on the thermal stability of HCP, the thermogravimetric analysis (TGA) of HCP with and without ultrasonic degradation was determined. Fig. 4 showed the TGA thermogram; all samples were presented weight loss at a temperature lower than 100 °C, which related to the evaporation of free and bound water [36]. The initial thermal degradation temperatures of HCP, U200, U400, U600 were 203.79, 216.25, 222.38 and 230.77 °C, respectively. In the range of 200–450 °C, the weight loss of all samples was increased due to the thermal decomposition of the polysaccharide. The weight loss of HCP, U200, U400, and U600 was 56.86, 49.98, 35.21, and 32.34 %, respectively. At the pyrolytic temperature increased to the third range from 450-600 °C, HCP presented a slow mass loss of about 20.62 %, while the ultrasonic degraded HCP showed less mass loss. These results proved that ultrasonic could improve the thermal stability of the polysaccharide due to the exposure of more hydrophilic groups by breaking the large structure of the polysaccharide chain. This hydrophilic group can form a strong hydrogen bond with water molecules, improving the ability of polysaccharides to retain water at higher temperatures. These findings were in the same trend as the results obtained by Wani et al. [37], who studied the characterization of the extracted pectin from pomelo peel via pulsed ultrasound-assisted extraction and acidic hot water extraction and found that the pectin produced with the assistance of ultrasonic presented highest thermal stability compare with the pectin produced by hot acidic water extraction.

#### 3.3.3 XRD

**Fig. 5:**
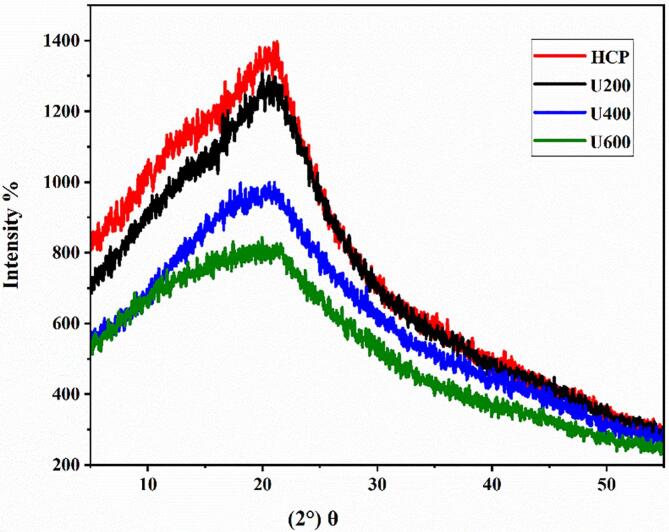
XRD spectra of HCP and ultrasonic degraded HCP.

The XRD pattern of HCP with and without ultrasonication presented the same broad diffraction peak near 20.91° as shown in Fig. 5, investigating that the native and ultrasonic degraded HCP had amorphous structures. The extension of the diffraction peaks of U200, U400, and U600 suggests a potential decrease in the grain of polysaccharides due to the ultrasonication degradation. Our findings were similar to those obtained by Liu et al. [23], who found that the ultrasonication degradation did not affect the crystallinity of the polysaccharide extracted from *Polygonum cyrtonema.*

#### 3.3.3 SEM

**Fig. 6:**
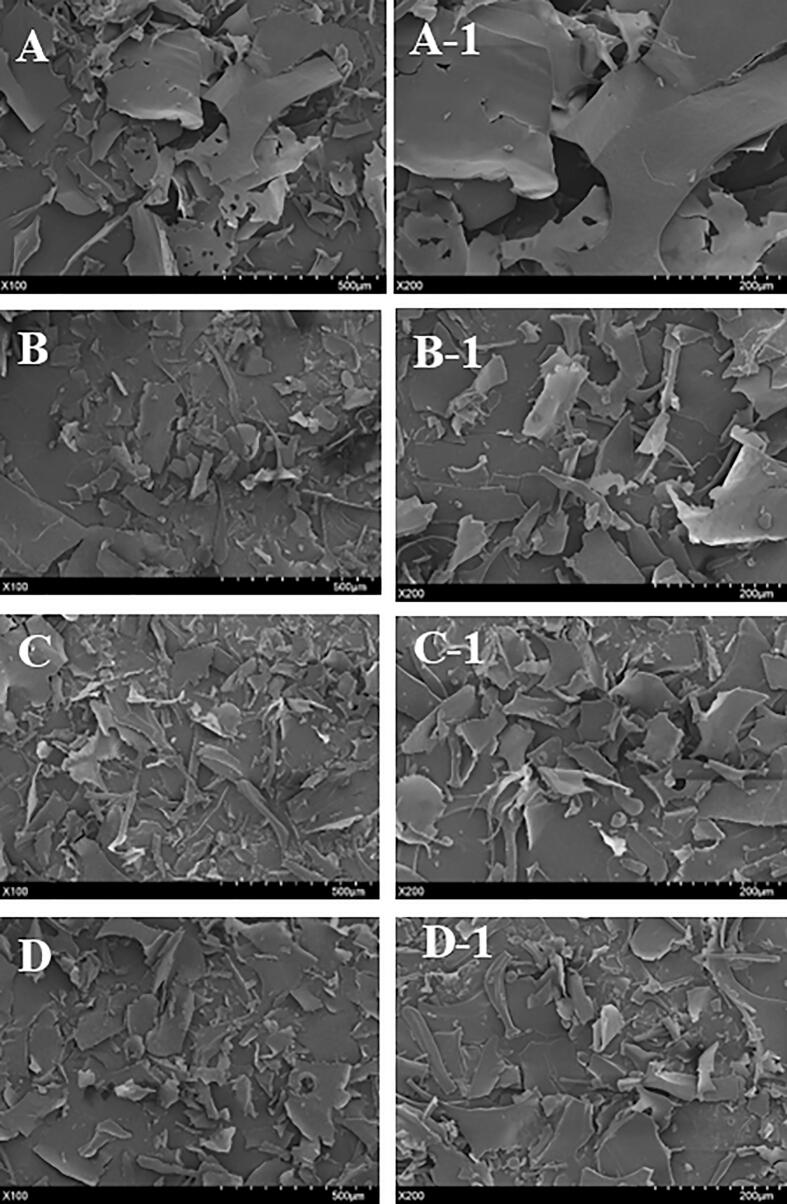
SEM image of HCP and ultrasonic degraded polysaccharide A: (magnification 100x, scale bar 500 µm) of HCP. A-1: (magnification 200x, scale bar 200 µm) of HCP. B: (magnification 100x, scale bar 500 µm) of U200. B-1: (magnification 200x, scale bar 200 µm) of U200. C: (magnification 100x, scale bar 500 µm) of U400. C-1: (magnification 200x, scale bar 200 µm) of U400. D: (magnification 100x, scale bar 500 µm) of U600. D-1: (magnification 200x, scale bar 200 µm) of U600.

SEM can be used to qualitatively analyze the surface morphology of polysaccharides. The morphological structure of HCP with and without ultrasonic was observed at 100x and 200x, as shown in Fig. 6. The surface morphology of all treatments was quite similar, exhibiting smooth and uniform surfaces. However, the surface area of ultrasonicated HCP decreased significantly compared to native HCP. This phenomenon could be related to the ultrasonication degradation that destroyed the aggregation in the polysaccharide [38]. These results may be attributed to collapsed cavitation bubbles forming a small concentrated area of high pressure, which is responsible for the disruption of glycosidase bonds [39].

#### 3.3.4 AFM

**Fig. 7:**
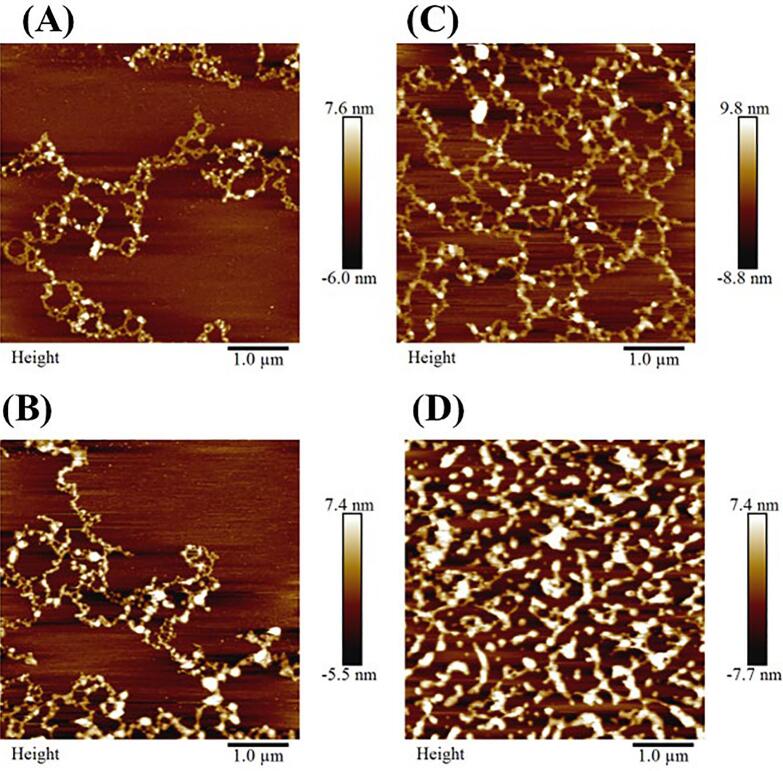
AFM image of HCP **(A)**, U200 **(B)**, U400 **(C)** and U600 **(D)**.

AFM is a widely used technique to characterize the morphological properties of polysaccharides [40]. The morphology of HCP, U200, U400, and U600 were imaged, as shown in Fig. 7. To assess HCP's confirmation changes and morphology after ultrasonic degradation. Fig. 7(A) shows the image of native HCP, in which the chain structure was visible with a flocculent structure and overlapped chain. After ultrasonication at 200 W, as shown in Fig. 7(B), the HCP chain appeared with highly lighter points, and the polysaccharide chain became a bit simpler than the native HCP. In the HCP treated with 400 W ultrasonic power, the polysaccharide chain was clearly visible with a simple structure, as figured in Fig. 7(C). For the HCP treated with 600 W ultrasonic power Fig. 7(D), the chain structure of HCP disappeared, shrank, and aggregated into a clumpy structure. Our findings suggested that, with the increase of ultrasonic power to degrade HCP, the polysaccharide chains were cracking gradually, and the aggregation of the polysaccharide chain was hindered, resulting in a uniform distribution. These results were on the same trend as obtained by Li et al. [41], who studied the effect of a combination of ultrasonic and hydrogen peroxide on the structure characterization of polysaccharides extracted from tremella fuciformis. Also, these findings were consistent with the result of SEM.

#### 3.3.4 Congo red analysis

**Fig. 8:**
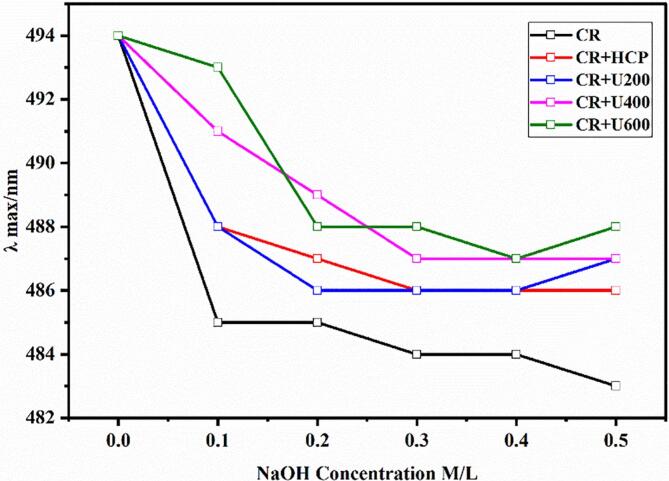
Maximum absorption (λ max) of Congo red and Congo red + HCP with and without ultrasonic degradation. (For interpretation of the references to colour in this figure legend, the reader is referred to the web version of this article.)

As shown in Fig. 8, the Congo red complex was formed by HCP with and without ultrasonic degradation. The red shift of λmax of all treatments was not observed in the alkaline range (0–0.5 M), indicating no triple helix structure in HCP before and after ultrasonic degradation. Moreover, some studies proved that a heteropolysaccharide could not form a triple helix structure [28,42]. The results of the monosaccharide composition of HCP demonstrated that HCP was heteropolysaccharide.

#### 3.3.4 NMR

**Fig. 9:**
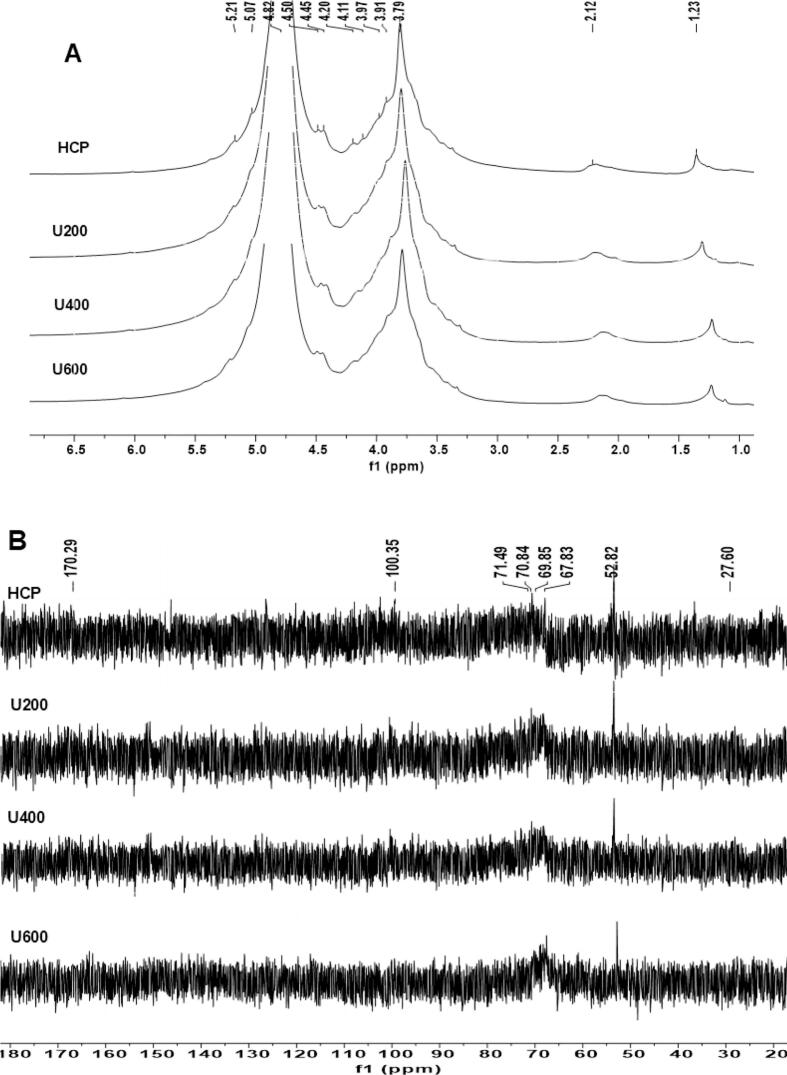
1H NMR spectra of HCP, U200, U400, and U600 (A). 13C NMR spectra of HCP, U200, U400, and U600 (B).

^1^H spectrum of HCP with and without ultrasonic treatment, as shown in Fig. 9(A), presented about 10 proton chemical shifts related to the α-(δ > 5.0 ppm) and β-(δ <5.0 ppm) polysaccharide configuration [43], there were *δ*5.21, 5.07, 4.82, 4.50, 4.45, 4.20, 4.11, 3.97, 3.91, and 3.79 ppm signals. As well as the chemical shifts at *δ* 2.12 and 1.23 ppm were methyl and acetyl proton chemical shifts. ^13^C spectrum of native and ultrasonic degraded HCP, as shown in Fig. 9(B), presented about 8 chemical shifts as follows: *δ*170.29, 100.35, 71.49, 70.84, 69.85, 67.83, 52.82, 27.60 ppm. The signal peak at 170.29 ppm related to C6 of 1,4α-D-GalA, the signals at 2.12 ppm and 4.11 related to H-2 and H-6 of 1,2-αRha, the signal at 52.82 confirmed the presence of methoxy group, whereas the acetyl H/C signal appeared at 1.23/27.60 ppm. The chemical shift at 5.07/100.35 ppm derived from H1/C1 of → 4) α −D-Glcp-(1 → [44]. The signals at 3.79/67.83,3.97/70.84 and 4.82/71.49 PPM is related to H2/C2, H3/C3 and H4/C4 of → 4) β −D-Glcp-(1→ [45]. The resonance signals in the HCP ^1^H NMR and ^13^C NMR spectra were congested and challenging to identify due to the large molecular weight of HCP, along with the presence of some monosaccharides in minimal quantities, which complicated the identification of all heteroatomic carbon signals. Additional literature has identified that the glycosidic linkage predominantly consists of 1,4-linked α-D-GalA and 1,2,4-linked α-L-Rha [46]. HBHP-3 polysaccharide was isolated and refined from *Houttuynia cordata*, with the predominant glycosidic bond being 1,4-linked α-D-GalA [7]. Therefore, *Houttuynia cordata* polysaccharides have different structures because of different places, extraction methods, and plant parts[4]. These results confirmed that the glycosidic linkage pattern was similar for ultrasonicated and native HCP, and ultrasonic degradation did not induce any changes in the main structure of HCP. Our findings were in the same trend as the result obtained by [14], who studied the effect of different ultrasonic treatments on the structure and bioactivities of blackcurrant polysaccharide and illustrated that the ultrasonic treatment did not affect the main structure of blackcurrant polysaccharide.

### 3.4 *In vitro* antioxidant activity

**Fig. 10:**
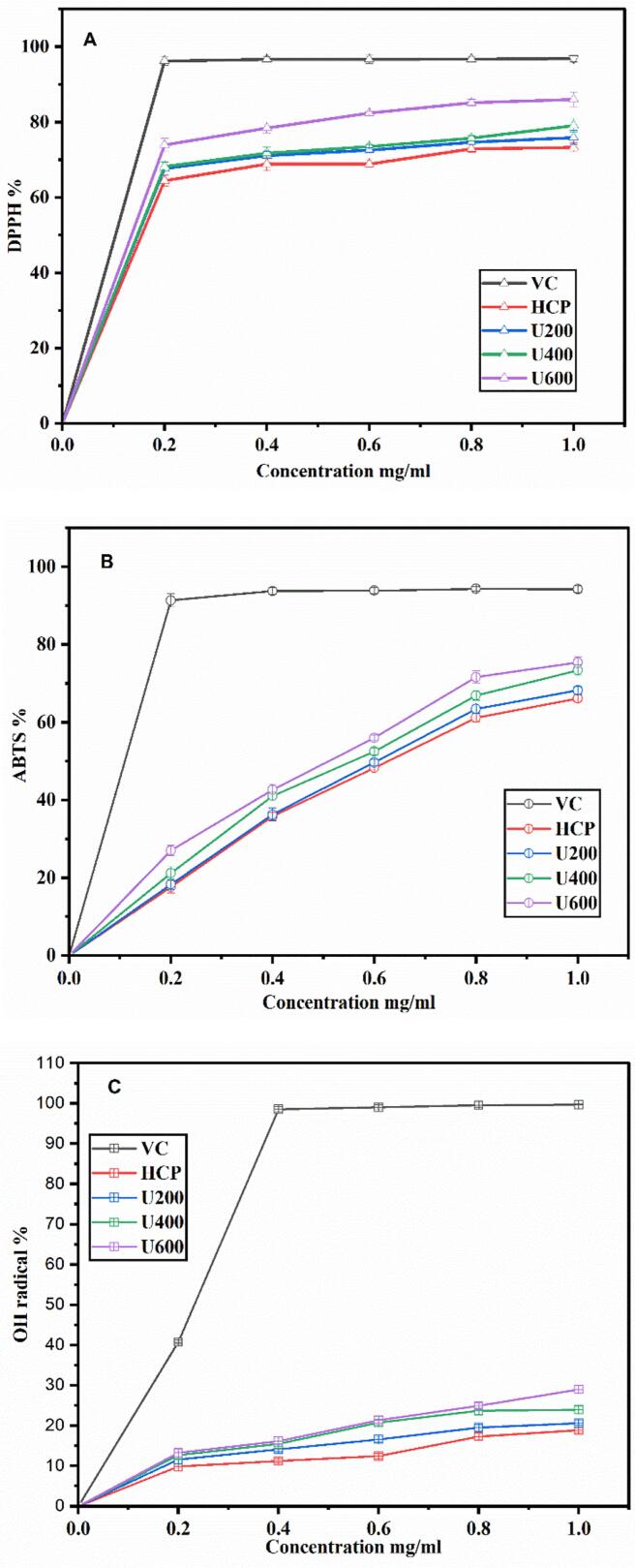
Antioxidant activity of HCP, U200, U400, and U600 in-vitro (A) DPPH radical; (B) ABTS radical; (C) hydroxyl radicle.

The antioxidant activity of HCP was determined via the radical scavenging activity assay, which is as follows: DPPH is a widely used radical for evaluating the antioxidant ability of different bioactive materials. As shown in Fig. 10(A), the scavenging activity of native HCP, ultrasonic degraded HCP, and V_c_ were increased with the test sample concentration. The ultrasonic degraded HCP presented higher scavenging compared with the native HCP, which was increased in order HCP< U200< U400<U600. At the highest tested concentration (1 mg/mL), DPPH scavenging activity was increased from 73.28 ± 1.25 % for native HCP to 85.98 ± 1.89 % for U600. The obtained result confirmed that ultrasonication degradation may expose more active moieties that could act as a hydrogen donor to scavenge the DPPH radicle.

As presented in Fig. 10(B), the ability of HCP, ultrasonic degraded HCP, and Vc to scavenge 2,2-azinobis-(3-ethylbenzothiazoline-6-sulfonic acid) radical was increased with the increasing concentration. Ultrasonic degraded HCP presented higher scavenging ability compared with native HCP, and the degraded HCP (U600) presented the highest scavenging activity (about 75.38 ± 1.38 %) compared with all treatments at concentration 1 mg/mL of HCP solution. These results proved that the greater the hydroxyl group produced by high ultrasonic intensity, the higher the scavenging activity against free radicals [47].

Hydroxyl radicals occur in the highest activity among reactive oxygen species, which can attack the biomolecules and cause oxidative damage to the cell. Scavenging activity of HCP and ultrasonicated HCP were increased in order HCP < U200 < U400 < U600, at the highest test concentration (1 mg/mL), they presented the maximum scavenging activity18.68 ± 0.62, 20.63 ± 0.81, 23.93 ± 1.0, 28.98 ± 0.88 %, respectively as shown in Fig. 10(C). The results indicated that U600 (the lower molecular weight polysaccharide) presented the more vigorous scavenging activity against hydroxyl radical. This was similar to the results reported by Chen et al. [48]; decreasing the molecular weight improves the antioxidant activity of polysaccharides. This phenomenon was in the same trend as Chen et al. [49], who studied the effect of ultrasonic degradation on the antioxidant activity of hawthorn pectin and proved that ultrasonic could increase the antioxidant activity of polysaccharides through DPPH, ABTS, and OH radicle. These results confirmed that ultrasonication could improve the antioxidant activity of polysaccharides due to some causes; on the one hand, the increase of monosaccharide glucuronic acid, xylose, mannose, rhamnose, glucose, galacturonic acid, galactose, and arabinose exposure on polysaccharide solution [50], which matched the result of the uronic acid content. On the other hand, the decrease in the polysaccharide molecular weight could be attributed to the contribution of more electrons to interact with the free radicle, resulting in better antioxidant activity [11,51].

### 3.5 *In vitro* hypoglycemic activity

**Fig. 11:**
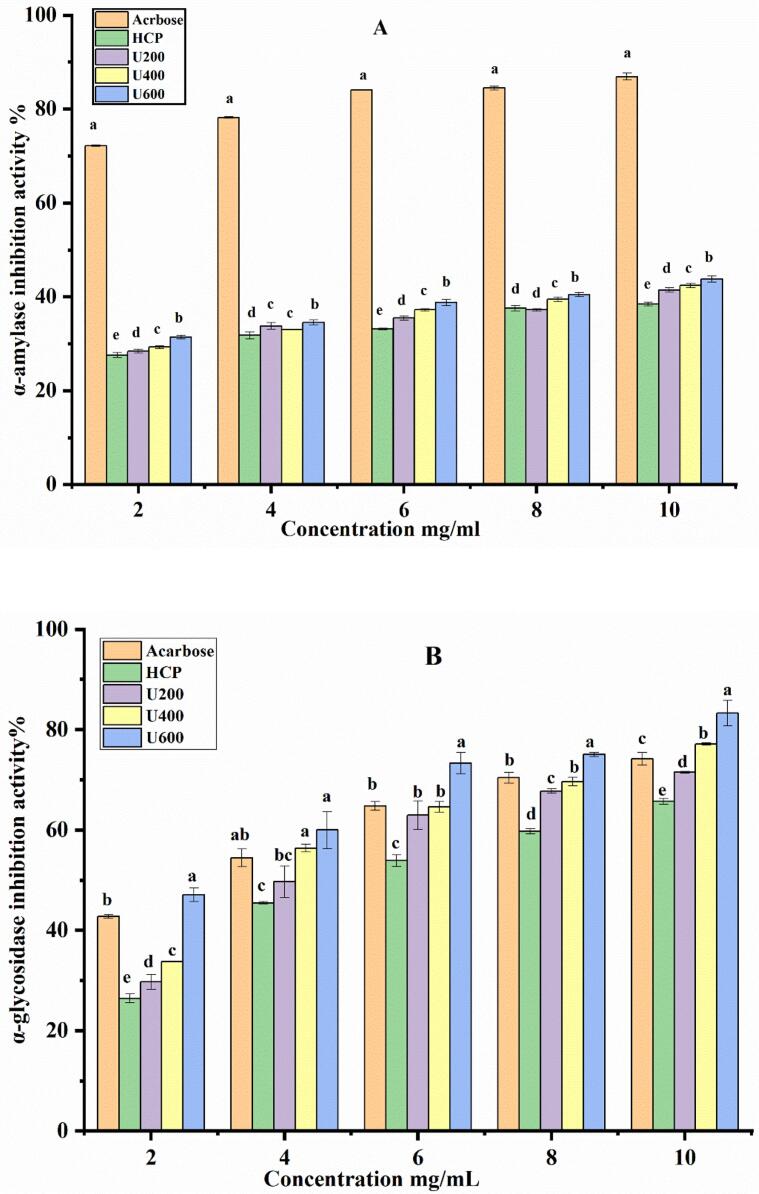
Hypoglycemic activity of HCP and ultrasonic degraded polysaccharide in-vitro. (A) α-amylase inhibition; (B) α-glycosidase inhibition.

Nowadays, inhibition of α-amylase and α- glycosidase enzymes have an interest as oral hypoglycemic agents for diabetics because of their ability to delay the absorption of glucose or fructose and delay the postprandial hyperglycemic [52,53]. Ultrasonication treatments have a valuable effect on enhancing the ability of HCP to inhibit α-amylase; as figured in Fig. 11(A), the inhibition was increased gradually with the increase of ultrasonication power, and the HCP treated with 600 W power presented the highest inhibition at about 43.80 ± 0.68 % compared with native polysaccharide 38.40 ± 0.53 % at concentration 10 mg/mL of HCP solution. The inhibition of the α-glycosidase enzyme was improved with ultrasonicated treatment, as presented in Fig. 11(B). The inhibition rate was increased with the increase of ultrasonication power. The HCP treated with ultrasonic 600 W presented the highest value of about 83.28 ± 2.56 %, and the native HCP inhibition value was about 65.67 ± 0.54 %. These results proved that ultrasonication could improve the inhibition of α-amylase and α-glycosidase activity, and degraded HCP may be considered an effective way to inhibit α-glycosidase, which counts as an essential enzyme for carbohydrate hydrolysis and could decrease the postprandial glucose level to control diabetes. Our findings were similar to the result obtained by Xu et al. [14], who studied the effect of ultrasound irradiation on the characterization and bioactivities of blackcurrant polysaccharide and found that ultrasonicated polysaccharide presented a higher inhibition rate against α-amylase and α-glycosidase compared with native blackcurrant polysaccharide.

## 4 Conclusion

In conclusion, the ultrasonic degradation induced a significant decrease in the molecular weight of HCP without significant structure changes compared with the native HCP. Notably, ultrasonic degradation enhanced HCP's physiochemical properties (Total polysaccharide content, uronic acid content solubility and thermal stability). Compared with the native HCP, ultrasonic degraded polysaccharide presented a vigorous antioxidant activity against (DPPH, ABTS, and OH) radicals. Moreover, the inhibition of α-amylase and α- glycosidase of HCP was improved with ultrasonic treatment. The ultrasonicated HCP with 600 W power (U600) presented the highest inhibition activity against α-amylase and α- glycosidase, about 43.80 ± 0.68 and 83.28 ± 2.56 %, which were higher than the native HCP inhibition activity of about 38.40 ± 0.53 and 65.67 ± 0.54 % at concentration of 10 mg/mL, respectively. The inhibition activity of U600 against α- glycosidase was higher than acarbose at the same concentration; the inhibition activity of acarbose only reached 74.15 ± 1.27 %, while U600 could reach 83.28 ± 2.56 % at a concentration of 10 mg/mL. These results proved that ultrasonication could be considered a green and efficient method for HCP degradation, improving its biological activities and increasing the utilization of polysaccharides in functional foods and pharmaceutical industries.

## CRediT authorship contribution statement

**Mohammed Mansour:** Writing – original draft, Methodology, Formal analysis, Conceptulization, Validation. **Ramy M. Khoder:** Writing – original draft, Data curation. **Lin Xiang:** Writing – original draft, Visualization. **Lan Lan Zhang:** Writing – original draft, Validation. **Ahmed Taha:** Writing – original draft, Validation, Software. **Alsadig Yahya:** Writing – original draft, Formal analysis. **Ting Wu:** Writing – review & editing, Data curation. **Hassan Barakat:** Writing – review & editing, Writing – original draft, Supervision. **Ibrahim Khalifa:** Writing – review & editing, Writing – original draft, Visualization. **Xu Xiaoyun:** Writing – review & editing, Supervision, Resources, Project administration, Funding acquisition.

## Declaration of competing interest

The authors declare that they have no known competing financial interests or personal relationships that could have appeared to influence the work reported in this paper.
